# The SARS-CoV-2 differential genomic adaptation in response to varying UVindex reveals potential genomic resources for better COVID-19 diagnosis and prevention

**DOI:** 10.3389/fmicb.2022.922393

**Published:** 2022-08-04

**Authors:** Naveed Iqbal, Muhammad Rafiq, Masooma, Sanaullah Tareen, Maqsood Ahmad, Faheem Nawaz, Sumair Khan, Rida Riaz, Ting Yang, Ambrin Fatima, Muhsin Jamal, Shahid Mansoor, Xin Liu, Nazeer Ahmed

**Affiliations:** ^1^Faculty of Life Sciences and Informatics, Baluchistan University of Information Technology, Engineering and Management Sciences (BUITEMS), Quetta, Pakistan; ^2^Department of Microbiology, Quaid i Azam University, Islamabad, Pakistan; ^3^Beijing Genomic Institute (BGI), Shenzhen, China; ^4^Department of Biological and Biomedical Sciences, Aga Khan University, Karachi, Pakistan; ^5^Department of Microbiology, Abdul Wali Khan University Mardan, Mardan, Pakistan; ^6^Agriculture Biotechnology Division, National Institute for Biotechnology and Genetic Engineering (NIBGE), Faisalabad, Pakistan

**Keywords:** SARS COVID-19, genomic adaptation, UV-solar radiation, COVID diagnosis, comparative genomics

## Abstract

Coronavirus disease 2019 (COVID-19) has been a pandemic disease reported in almost every country and causes life-threatening, severe respiratory symptoms. Recent studies showed that various environmental selection pressures challenge the severe acute respiratory syndrome coronavirus-2 (SARS-CoV-2) infectivity and, in response, the virus engenders new mutations, leading to the emergence of more virulent strains of WHO concern. Advance prediction of the forthcoming virulent SARS-CoV-2 strains in response to the principal environmental selection pressures like temperature and solar UV radiation is indispensable to overcome COVID-19. To discover the UV-solar radiation-driven genomic adaption of SARS-CoV-2, a curated dataset of 2,500 full-grade genomes from five different UVindex regions (25 countries) was subjected to in-depth downstream genome-wide analysis. The recurrent variants that best respond to UV-solar radiations were extracted and extensively annotated to determine their possible effects and impacts on gene functions. This study revealed 515 recurrent single nucleotide variants (rcntSNVs) as SARS-CoV-2 genomic responses to UV-solar radiation, of which 380 were found to be distinct. For all discovered rcntSNVs, 596 functional effects (rcntEffs) were detected, containing 290 missense, 194 synonymous, 81 regulatory, and 31 in the intergenic region. The highest counts of missense rcntSNVs in spike (27) and nucleocapsid (26) genes explain the SARS-CoV-2 genomic adjustment to escape immunity and prevent UV-induced DNA damage, respectively. Among all, the most commonly observed rcntEffs were four missenses (RdRp-Pro327Leu, N-Arg203Lys, N-Gly204Arg, and Spike-Asp614Gly) and one synonymous (ORF1ab-Phe924Phe) functional effects. The highest number of rcntSNVs found distinct and were uniquely attributed to the specific UVindex regions, proposing solar-UV radiation as one of the driving forces for SARS-CoV-2 differential genomic adaptation. The phylogenetic relationship indicated the high UVindex region populating SARS-CoV-2 as the recent progenitor of all included samples. Altogether, these results provide baseline genomic data that may need to be included for preparing UVindex region-specific future diagnostic and vaccine formulations.

## Introduction

In December 2019, clusters of pneumonia cases were reported from the Wuhan city, Hubei province, China. Some of the early disease cases were reported working in the live animal market. On 11 March 2020, the WHO announced the disease outbreak, now named coronavirus diseases 2019 (COVID-19), as a public health emergency of international concern and declared it a pandemic (Koyama et al., 2020). As of June 2022, ~ >528.82 million positive cases were reported to WHO across the world [WHO Coronavirus (COVID-19) Dashboard, 2022], with more than 6.29 million deaths. The COVID-19 symptoms range from mild fever, cough and fatigue to severe shortness of breath, and loss of taste and smell (Guan, 2020; Wang D. et al., 2020), with the 5% average fatality rate of all confirmed positive cases, which is of lower than SARS-CoV (9.6%) and MERS (34.3%) (World Health Organization., 2003, 2019; Wang C. et al., 2020).

After the preliminary etiological investigations based on the exclusion of all common respiratory pathogens, the deep meta-transcriptomic sequencing of the patient's bronchoalveolar lavage fluid revealed the abundance of a viral strain from β-coronavirus (CoV) genus (Shi et al., 2016, 2018; McMullan et al., 2019; Yadav et al., 2019; Abdelrahman et al., 2020; Wu et al., 2020). The COVID-19-causing virus showed 89.1%, 79.5%, and 50% sequence homology to previously reported SARS-like coronavirus strains, namely, bat SL-CoVZC45, SARS-CoV, and MERS, respectively (Wang et al., 2015; Wu et al., 2020). Based on the sequence homology to SARS-like viruses, the crown-like viral structure, and the consequent manifestation of severe respiratory disease symptoms, the COVID-19-causing virus is designated as SARS-CoV-2 (severe acute respiratory syndrome coronavirus-2) (Lu et al., 2020; Wu et al., 2020). Furthermore, most SARS-like coronaviruses have been identified in bats (Hamre and Procknow, 1966; McIntosh et al., 1967; Li et al., 2005), and the SARS-CoV-2 shares 100% amino-acid sequence similarity with NSP7 and E protein of the bat SARS-like coronavirus strain (bat SL-CoVZC45) (Wu et al., 2020). These findings suggest that bats are the possible natural reservoirs for most SL-CoVs, including SARS-CoV-2.

The SARS-CoV-2 genomic characterization revealed 29,903 nucleotide long single-stranded positive-sense RNA (ribonucleic acids) comprising a multi-domain nonstructural protein (NSP) encoding ORF1ab, four structural protein genes (spike “S,” envelope “E,” membrane “M,” and nucleocapsid “N”), and six accessory protein-encoding genes (ORF2a, ORF6, ORF7a, ORF7b, ORF8, and ORF10) (Koyama et al., 2020). The SARS-CoV-2 was found capitalizing its spike structural protein for host cell (respiratory epithelial) attachment and subsequent entries *via* the angiotensin-converting enzyme 2 (ACE2) receptor (Hoffmann et al., 2020).

Since December 2019, whole-genome sequence analysis revealed hundreds of viable genetic variants of SARS-CoV-2 from different parts of the globe. Within SARS-CoV-2, the observed predominating drivers of genetic variation are the single-nucleotide variants (SNVs) caused by the error-prone viral polymerases (Smertina et al., 2019; Lu et al., 2022) and endogenous mutagenesis *via* the host RNA-editing enzymes (nucleotide deaminases APOBEC: C>U and ADAR: A>G) (Placido et al., 2007; Moris et al., 2014; Mourier et al., 2021; Tong et al., 2022). The genome-wide studies of large sets of SARS-CoV-2 revealed SNV-based nucleotide substitution rate of ~1 × 10^−3^ per year (Duchene et al., 2022), closer to the 1.42 × 10^−3^ Ebola virus substitution rate reported from West Africa during 2013–2016. However, SNVs are not the only genetic variations discovered in coronaviruses, but the small insertions/deletions of viral or non-viral sequences were also reported in various genetic variants of coronavirus genomes possibly caused by the discontinuous nature of viral transcriptase for sub-genomic mRNA synthesis (Licitra et al., 2013; V'kovski et al., 2021). In total, a large proportion of the mutations represent neutral “genetic drift” or have died out quickly, and a small subset is affecting viable viral traits, such as host range, transmissibility, antigenicity, pathogenicity, and adaptability of the virus to various selection pressures.

Various biotic and abiotic selection pressures challenge the SARS-CoV-2 persistence, transmission, infectivity, host cell entry efficacy, and pathogenesis (Pica and Bouvier, 2012). Since RNA viruses, *via* high mutation rate, have demonstrated a great potential for rapid evolution and adaptation to new environmental conditions in the absence of a proper proofreading RNA polymerases activity (Holland et al., 1982; Rubio et al., 2013). Therefore, to escape stress conditions, the coronaviruses continuously engender new genomic variations, potentially resulting in the emergence of more virulent SARS-CoV-2 strains of WHO concern with higher transmission and mortality rates (Sanjuán and Domingo-Calap, 2016; Chin et al., 2020; Koyama et al., 2020; Seyer and Sanlidag, 2020; Kumar et al., 2021; Soh et al., 2021). The commonly experienced biotic selection pressures in human hosts may include natural immunity (Clapham et al., 2020), host genetic makeup (COVID-19 Host Genetics Initiative, 2021), monoclonal antibodies produced in response to vaccines (Rella et al., 2021; Shah et al., 2021), antiviral drugs, and convalescent sera, whereas solar radiation (Chiyomaru and Takemoto, 2020) (ultraviolet radiations) (Seyer and Sanlidag, 2020), temperature (Chin et al., 2020; Wang J. et al., 2020), relative humidity (Ahlawat et al., 2020; Ghoushchi et al., 2020), and air pollutants (Coccia, 2020) are the widely studied abiotic selection pressures on viral populations (Tan et al., 2005; Shaman et al., 2010; Otter et al., 2016; Chattopadhyay et al., 2018; Dalziel et al., 2018; Gardner et al., 2019). Studies revealed a negative correlation between the environmental conditions (temperature and humidity) and the H3N2 strain of the influenza flu virus (Lowen et al., 2007; Reich et al., 2019). Additionally, ultraviolet radiation imposed negative selection pressure on strains of influenza and related coronaviruses (Darnell et al., 2004), and more recently, Ratnesar-Shumate et al. showed that the UV-solar radiation induced SARS-CoV-2 nucleic-acid damage and subsequent viral inactivation (Ratnesar-Shumate et al., 2020).

Predicting genomic level adaptation of SARS-CoV-2 in response to various selection pressures is indispensable in understanding the viral spread, mutation, pathogenicity, control, and future treatment options to effectively tackle COVID-19 (O'Reilly et al., 2020). Solar ultraviolet radiation is thought to have a great impact on the formation of viral populations by selecting variants that can withstand UV-solar radiations (Ratnesar-Shumate et al., 2020). In this study, to investigate the SARS-CoV-2 genomic adaptation in response to UV solar radiation, we analyzed 2,500 high-quality, full-length genomes from five different WHO's defined UVindex regions. The comparative genome-wide analysis of SARS-CoV-2 populations revealed differential genomic adjustments in response to different ultraviolet solar radiations. All identified differential genomic signatures in response to various UVindex ranges provide baseline data for future more effective molecular COVID-19 diagnosis and region-specific vaccine production against COVID-19.

## Methods

### Sampling

In this study, to reveal the genomic adaptation of SARS-CoV-2 in response to UV-radiation, all COVID-19 experienced countries, which have uploaded at least 100 full-length, high-quality SARS-CoV-2 genomes, are included. Based on the WHO and US-EPA ultraviolet (UV) radiation exposure categories (Table 1), all included countries are divided into the following five groups according to their respective ultraviolet index (UVindex) records (World Health Organization, 2002; Fioletov et al., 2004). Low UVindex countries (UVindex range: <2), Moderate UVindex countries (UVindex range: 3–5), High UVindex countries (UVindex range: 6 to 7), Very_High UVindex countries (UVindex range: 8–10), and Extreme UVindex countries (UVindex range: >11).

**Table 1:** Ultraviolet radiation exposure categories by WHO UVindex guide.

**Exposure categories**	**UVindex range**
Low	≤ 2
Moderate	3–5
High	6–7
Very high	8–10
Extreme	≥11

*Each row is filled with different shade using the WHO assigned color for each UVindex range*.

UVindex mean data for 12 months (from 7 December 2020 to 8 December 2021) for all included countries were obtained from the monthly weather forecast and climate by WeatherAtlas (retrieved on 08 December 2021, at 15:30 GMT/UTC + 5h; https://www.weather-atlas.com/). The UVindex value for each country was presented as a single value rounded to the nearest whole number. For each category, irrespective of the country's geographical location, the most relevant (top of the category's list) five countries were selected provided that the country experiencing UVindex falls in the specified category range and must have at least 100 full-length, high-quality genome sequences reported in publicly accessible databases (Supplementary Table 1). Initially, for all UVindex categories, the all available (total of 8,631) full-length SARS-CoV-2 genomes were downloaded from GISAID on 11 December 2021, GenBank on 15 December 2021, the Chinese National Genomics Data Center Genome Warehouse on 23 December 2021, and the Chinese National Microbiology Data Center on 23 December 2021 (Benson et al., 2012; Shu and McCauley, 2017; CNCB-NGDC Members and Partners, 2021). To process high-quality, full-length genomes in each of the UVindex category, downloaded sequences shorter than 29,700 bps and containing seven consecutive ambiguous nucleotides (NNNs) were excluded from the downstream analysis. The China National Center for Bioinformation annotations was used to remove redundancy (Gong et al., 2020). Downloaded sequences containing 50 ambiguous bases were removed from the downstream analysis to reduce the number of false-positive variants using Trimmomatic version 0.39 (Bolger et al., 2014). Finally, using the accustomed Perl script, a 100 high-quality genome sequences from each of the five included countries in a UVindex category were randomly selected, so in a nutshell, for all five UVindex categories, 2,500 full-length SARS-CoV-2 reported genomes were retained for analysis.

### Reference genome

The SARS_CoV-2 (NC_045512.2) sequence was used as a reference genome in this study. The NC_045512.2 was sequenced in December 2019 from a sample recovered from Wuhan, China (Wu et al., 2020). According to the standard procedure for variant detection (DePristo et al., 2011), to retrieve high-quality variants, first, each sample was converted to short FastQ reads using emboss-splitter (Rice et al., 2000) and an accustomed fasta-to-fastq.pl script available in GitHub (Dabbish et al., 2012).

### Read mapping

High-quality reads from each sample were mapped to the latest available reference SARS-CoV-2 genome NC_045512.2 using the BWA-MEM algorithm with the default minimum seed length of 20, gap open penalty 6, gap extension penalty 1, and matching score 1 (Li, 2013). For variant identification and downstream processing, open-source software packages were used. The “RealignerTargetCreator” and “InDelRealigner” command-line tools of the Genome Analysis Toolkit (GATK version 3.3.0) were used to fix all mapping issues through locally realigning improperly mapped reads, possessing variant artifacts at their terminals (McKenna et al., 2010). Before calling variants, Picard, Samtools, and BWA were used to generate the reference and bam file indexes (Li and Durbin, 2009; Li et al., 2009; McKenna et al., 2010; DePristo et al., 2011).

### Variant calling and quality filtration

Any deviation of the properly mapped read sequence to the reference genome NC_045512.2 was called as a variation. For variant discovery, initially, the “mpileup” utility of bcftools, with default parameters, was used to call genotypes for each of the samples included in this study. From the derived genotypes, high-quality variants were identified as any deviation of the mapped read sequences from the reference genome using the bcftools “call” command (Li, 2011). To differentiate between real hereditary variants from the false-positive data-processing artifacts (caused ambiguous bases), a calibrated statistical likelihood was generated for each of the identified variant loci using the GATK “Variant Recalibrator” and “ApplyRecalibrator” functions (McKenna et al., 2010). Finally, false-positive data-processing artifacts were removed using the following options of bcftools filter and GATK variant filtration; (a) variants were removed with a Phred quality score ≤ 20; (b) since Fisher's exact test-based Phred-scaled *P*-value (FS) represents strand bias for the reference and alternative allele, a sign for the false-positive variant. Therefore, variants with FS values >60 were filtered out from the downstream analysis (Kim et al., 2017; Iqbal et al., 2019).

### Variant functional annotation and prioritization

After filtration, high-quality variants were retained for each of the UVindex categories. Furthermore, high-quality variants to predict possible variant functional effects, impact, and their respective distribution across the reference NC_045512.2 genome were comprehensively investigated. The SnpEff_4.3 was used to attribute each variant by a functional class and offered various annotation levels to identify potential coding variants. For functional annotation, the SnpEff database was developed according to the SnpEff database building protocol (Cingolani et al., 2012) using the NCBI SARS-CoV-2 sequence annotation resources (NC_045512.2; Bio-Project, PRJNA485481; https://www.ncbi.nlm.nih.gov/sars-cov-2/). For all potential coding variants, the assigned SnpEff functional class vocabularies were UTR 3 prime, UTR 5 prime, splice site donor, splice site acceptor, splice site region, downstream, upstream, disruptive in-frame deletion and insertion, and conserved in-frame insertion and deletion. The results are provided in the list of functionally annotated variants (Supplementary Material: rcntSNV_UVindex.snpEff.vcf). A customized script was developed in Python to extract all identified variants for each of the genes in all UVindex categories (Supplementary Material: rcntSNVs_genes_functional_effects_UV.Case.genes). Following variant functional annotation, all coding region variants were compared to find UVindex category-specific and overlapping variants using vcftools (Danecek et al., 2011), the bioinformatics, and evolutionary genomics resources (http://bioinformatics.psb.ugent.be/webtools/Venn/).

### Phylogeny

For phylogeny, sequences were precisely chosen with <30 variations, and the lengths were adjusted by 5′ UTR and 3′ UTR truncation, without losing the key sequence sites. From this sequence pool, for an optimal phylogenetic relationship, a subset of 125 high-quality SARS-CoV-2 whole-genome samples (25 from each of the UVindex category) randomly selected in Perl by using a random number generator. All selected genomes were first aligned using the progressive multiple sequence alignment method of ClustalW (Thompson et al., 1994). The MEGA X (version 11.0.10) was used to produce and visualize the phylogenetic tree (Kumar et al., 2018). The maximum likelihood approach with Tamura-Nei substitution model, uniform rates among sites, all sites' data treatment, 1,000 bootstrap value, and nearest neighbor interchange (NNI) heuristic method was used for the best interfacing of a tree.

## Results and discussions

To determine the differential genomic adaptation of SARS-CoV-2 in response to different UVindex ranges, 2,500 full-length, high-quality reported genomes were investigated from 25 countries, classified into five distinct categories based on the country's UVindex exposures. UVindex-based categories are described in the “Methods” section (Table 1). A total of 500 full-grade genomes were included from each of the defined UVindex-based categories; for the Low UVindex category, genomes were obtained from Estonia, Faroe Islands, Iceland, Norway, and Sweden; for the Moderate UVindex category, genomes were retained from Kazakhstan, North Macedonia, South Korea, Spain, and Georgia, the United States; for the High UVindex category, genomes were maintained from Cyprus, Iran, Japan, New Zealand, and Florida, the United States; for the Very_High UVindex category, genomes were acquired from Bahrain, Bangladesh, Egypt, Kuwait, and Saudi Arabia; and for the Extreme UVindex category, genomes were included from Brazil, Ecuador, Singapore, Suriname, and Uganda (Supplementary_info_file.docx, Supplementary Table 1, and for geographical location, please see the map from Supplementary_map1, Supplementary Material). Accustomed Perl script was used to randomly select 100 high-quality SARS-CoV-2 genomes from each of the included countries.

### Variant discovery (total/rcntSNVs)

**Figure 1:**
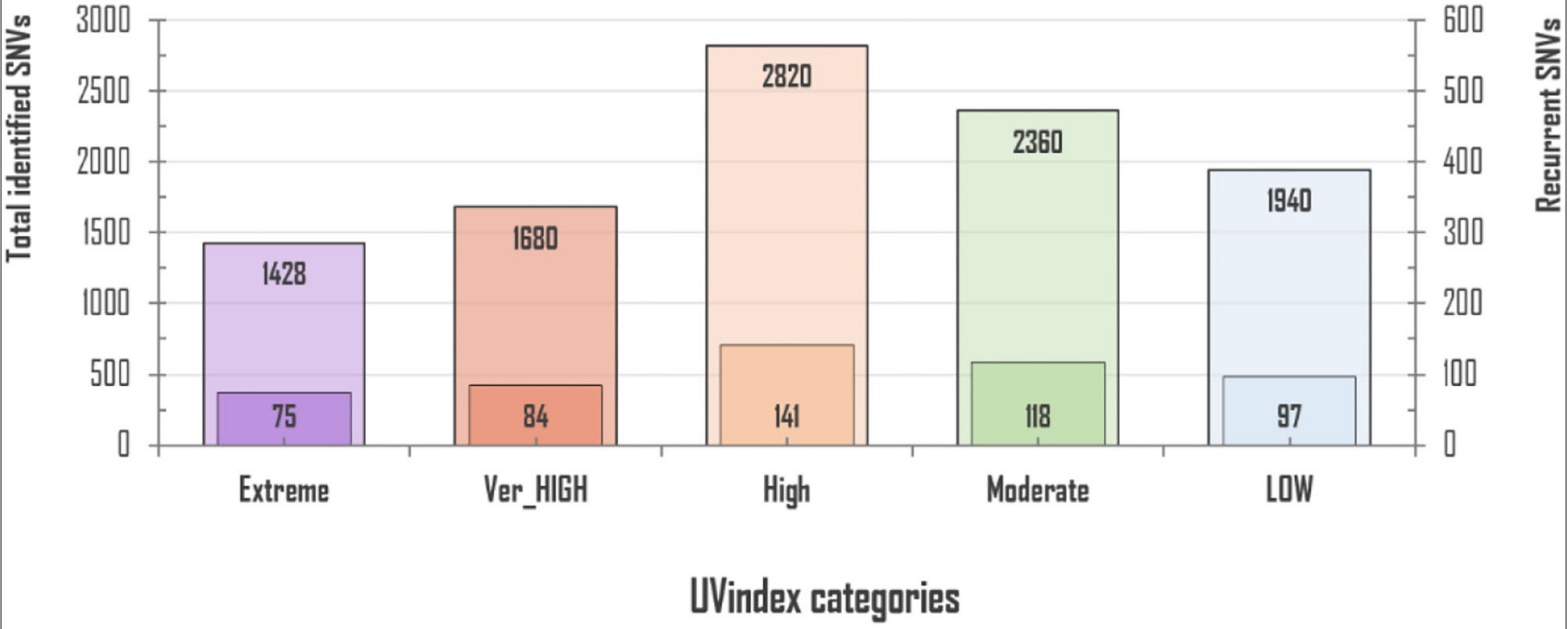
Total and recurrent SNVs (rcntSNVs) count in all examined 2,500 SARS-CoV-2 genomes, grouped in five distinct UVindex-based categories. For each WHO's defined UVindex category, the outer bar represents total identified SNVs, whereas the inner short bar represents predicted rcntSNVs.

For 2,500 SARS-CoV-2 complete genome samples, we discovered a total of 10,228 single nucleotide variants (SNVs) with an average variation load of one SNV after every 15.49 nucleotides per UVindex category (averaging ~3.92 SNVs/sample). In each UVindex category, countries are included based on their commonly shared UVindex ranges, irrespective of their relative humidity, temperature, altitude, geographical location, and many other selection pressures. Considering our sampling strategy, all identified SNVs in each UVindex category are the probable genomic adjustments against all experienced biotic and abiotic selection pressures, whereas only the most common SNVs in a UVindex category are the potential genomic adaptation of SARS-CoV-2 in response to UVindex. Therefore, based on a 25% reoccurrence rate in a UVindex category, a sum of 515 (5.03% of a total of 10,228) recurring SNVs were carefully prioritized to discover the SARS-CoV-2 genomic responses to a commonly experienced environmental selection pressure, the UV solar radiation. These SNVs with atleast 25% reoccurrences in each UVindex category are termed recurrent-SNVs (rcntSNVs). For all UVindex categories, lists of all discovered rcntSNVs are given in Supplementary_info_file.docx Supplementary Tables 2–6. Of the total, the least number of rcntSNVs (75) were observed in SARS-CoV-2 genomes included from countries exposed to Extreme UVindex solar radiation, revealing that the Extreme UVindex solar radiation employs negative selection pressure by damaging viral DNA and thus limits the diversity of SARS-CoV-2 strains. Our finding is consistent with the hypothesis that Extreme UVindex radiations induces viral DNA damage to disinfect the SARS-CoV-2 without altering its morphology (Lo et al., 2021). Furthermore, the solar UV radiation of extreme intensity inactivates SARS-CoV-2 and other related strains of corona and influenza viruses on surfaces (Pi et al., 2003; Darnell et al., 2004; Ianevski et al., 2019; Ratnesar-Shumate et al., 2020). On the contrary, the highest number of rcntSNVs (141) was discovered in the High UVindex region, suggesting that the large majority of SARS-CoV-2 variants/strains are adapted to High UVindex solar radiation. A. Ianevski et al. also showed the highest counts for the active influenza virus strains populating High UVindex experiencing parts of Northern Europe from 2010 to 2018 (Ianevski et al., 2019). Based on these findings, we propose that COVID-19-causing viruses have had sufficient evolutionary time to acquire genomic-level adaptation in High UVindex regions, probably in their primary natural reservoir (bat). Our findings are scientifically in line with the Li et al.'s work that found bats families, being the zoonotic origin of several SARS-like coronaviruses, greatly enriched in tropical regions experiencing High UVindex solar radiations (e.g., Guangdong, Guangxi, Hubei, and Tianjin) (Hamre and Procknow, 1966; McIntosh et al., 1967; Li et al., 2005; Wu et al., 2020). Figure 1 shows the total number of identified and rcntSNVs in each of the UVindex category.

### rcntSNVs genomic distribution

**Figure 2:**
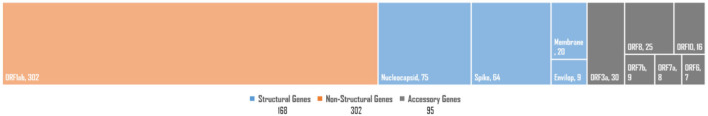
The SARS-CoV-2 genome-wide distribution of all observed high-quality rcntSNVs. Structural protein-encoding genes category is shown in orange (left-most), non-structural protein-encoding genes category is represented in blue (in the middle), whereas accessory genes category is shown in gray blocks (right-most). In each category, the smaller blocks and their sizes represent genes in a particular category and their respective rcntSNVs load, respectively.

The SARS-CoV-2 genome exhibits two non-structural multi-domain protein-encoding genes (ORF1a and ORF1b), four structural protein-encoding genes (SPeGs; S, E, M, and N), and up to six genes that encode accessory proteins 3a, 6, 7a, 7b, 8, and 10a (Brant et al., 2021). Our in-depth analysis for gene-set-based distribution of all potentially UVindex responding variants revealed the large majority of the total rcntSNVs (302: 53.45%) in the non-structural protein-encoding genes (ORF1ab), followed by 168 (29.73%) in four SPeGs (N = 75, S = 64, M = 20, and E = 9), whereas only 95 (16.81%) were found in six accessory genes (Figure 2). These inferences are in agreement with the genomic architecture of the SARS-CoV-2 (Wu et al., 2020) and illustrate that SARS-CoV-2 has done most (approximately >53%) of the genomic-level adaptation in non-structural multi-domain protein-encoding genes (ORF1ab) to adapt various UVindex regions, where the accessory protein-encoding genes were the most conserved gene-set of SARS-CoV-2.

**Figure 3:**
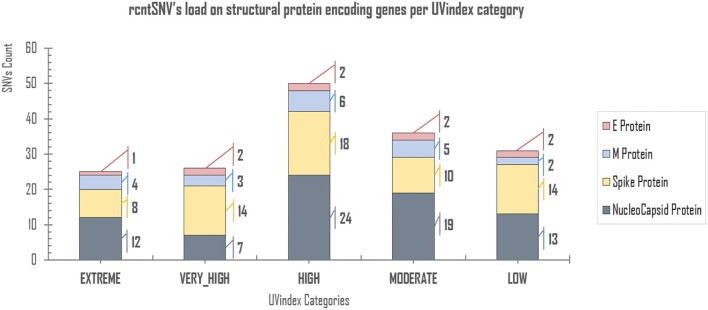
rcntSNVs load on structural protein-encoding genes per UVindex category. Each UVindex category is represented by a stacked column, whereas the bars in gray, yellow, blue, and pink represent numbers of recurrent SNVs in nucleocapsid (N), spike (S), membrane (M), and envelope (E) structural protein-encoding genes, respectively. For each UVindex categories, the rcntSNVs count for all proteing-encoding genes are given on the right-hand side of stacked-bars.

Of all the virion proteins, the structural gene products were directly exposed to environmental selection pressures, like solar UV radiation. Therefore, the downstream analysis was focused to identify rcntSNVs in E, M, S, and N SPeGs for each of the UVindex category (Figure 3). Of the total identified 168 structural rcntSNVs, we discovered 75, 64, 20, and 9 in nucleocapsid, spike, membrane, and envelope SPeGs, respectively. Of all four SPeGs, the nucleocapsid gene has gone through most of the genomic rearrangements, possibly to shield the nucleic acid damaging effects of UV radiation *via* adaptation in response to differential UVindex exposures. These findings support recent studies on SARS-CoV-2, revealing the adverse effects of UV radiation (UVC) on nucleic acid without affecting viral proteins (Chang et al., 2014), and the nucleocapsid protein's key role in packaging and protecting COVID-19 viral genome in a viable virion (Tahara et al., 1994, 1998; Lai and Cavanagh, 1997).

### rcntSNVs functional effects

**Figure 4:**
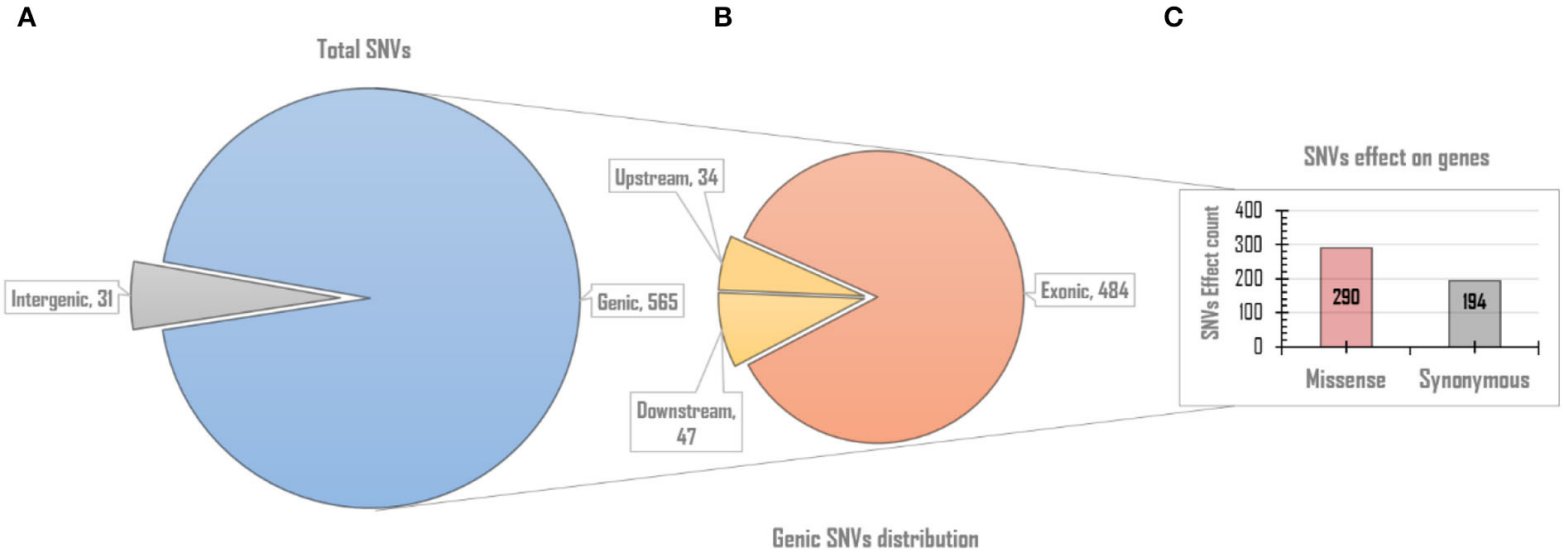
The overall genomic and functional effect-based distribution of all identified rcntSNV-effects (rcntEffs). **(A)** Displays the distribution of all predicted rcntEffs into coding/genic and non-coding/intergenic regions. **(B)** Upon further in-depth annotation, the genic region rcntEffs are distributed among protein-coding (exons) and gene-regulatory (up/downstream) regions of all SARS-CoV-2 genes, whereas the bar chart **(C)** represents the total missense and synonymous functional effects counts exhibited by all identified rcntEffs found segregating in the gene's exonic regions.

Since rcntSNVs in each of the five UVindex categories best represent differentially adapted SARS-CoV-2 populations. Therefore, all rcntSNVs were functionally annotated to predict their direct effects and impacts on the genes' functions. One SNV may have more than one effects, possibly due to the gene overlapping (Cingolani et al., 2012; Iqbal et al., 2019). As a result, slightly more rcntSNV-effects (rcntEffs) were observed compared to the total rcntSNV count. In this study, a total of 596 functional rcntEffs were discovered for all rcntSNVs. Functional annotation revealed only 31 (5.2%) rcntEffs in the non-coding intergenic regions, and the remaining 565 (94.8%) were located in the genic regions of the SARS-CoV-2 genome. Of the total genic region rcntEffs, 81 (14.3%) were detected in the gene's regulatory regions, positioned 200 bp upstream (34 count) and downstream (47 count) of all genes, and the remaining 484 (85.7%) were found in the coding regions (exonic). These results are scientifically in line with the genomic architecture of the SARS-CoV-2, and similar results were also shown by Koyama et al. (2020). The overall functional rcntEffs count for all rcntSNVs and their corresponding distribution across the SARS-CoV-2 genome are shown in Figure 4.

**Figure 5:**
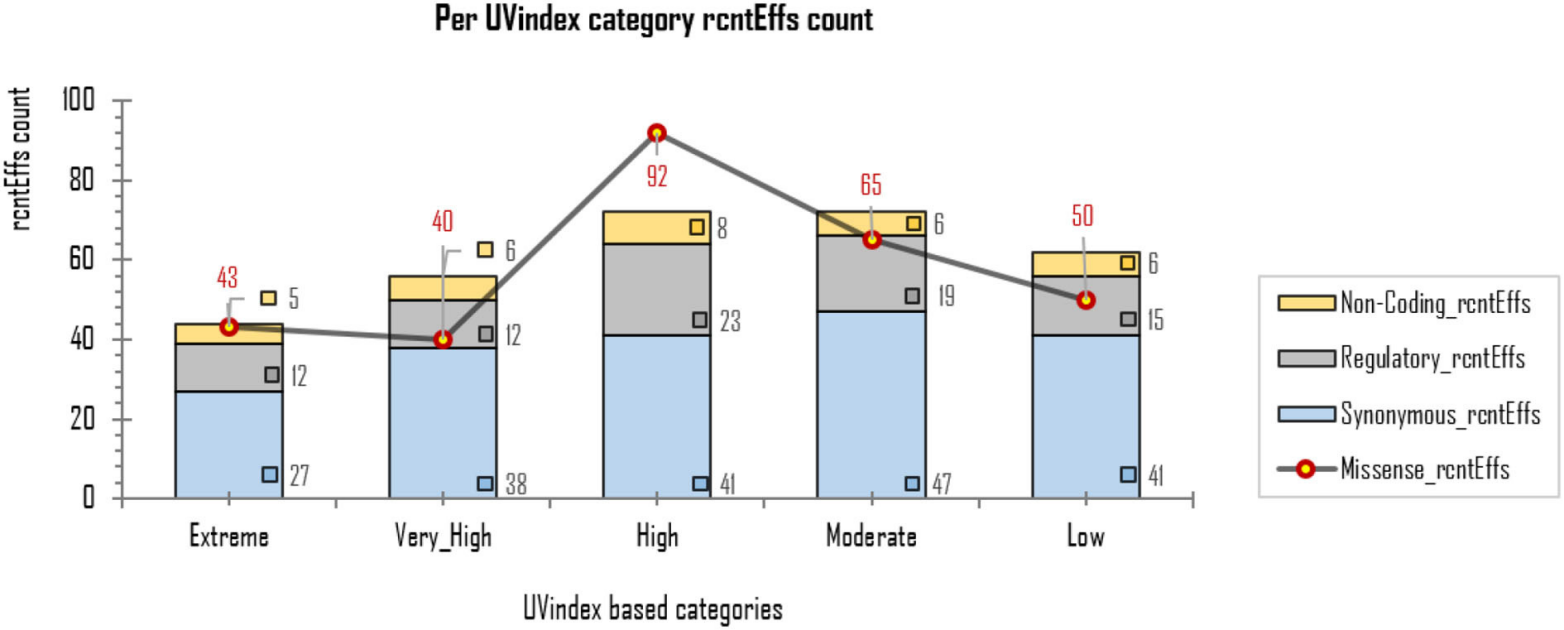
Different functional effects (rcntEffs) predicted for all rcntSNVs in all five WHO's defined UVindex categories are shown using a combo bar-line chart. The most prevalent rcntEffs missense are displayed using red-pointed gray line, whereas the synonymous, regulatory, and non-coding rcntEffs are represented here in blue-, gray-, and yellow-stacked columns, respectively.

The exonic rcntEffs set comprises 290 missenses and 194 synonymous genes' functional effects. Interestingly, of the total identified rcntSNVs in all UVindex categories, the highest number of the variants are with missense functional effects (290; 48.7%), suggesting that in response to immense selection pressure imposed by varying degrees of UV radiation, the SARS-CoV-2 has capitalized on the high impact missense variation enrichment to qualify for UV radiation stress. More than 71.38% (~207) of the total missense rcntEffs are found segregating in High (92; 31.7%), Moderate (65; 22.4%), and Low (50; 17.2%) UVindex categories. Suggesting that the UVindex range ≤ seven allows more SARS-CoV-2 strains to survive. On the contrary, the UVindex ≥ eight imposes strong negative selection pressure on SARS-CoV-2 as only ~28.62% (83) of the total missense rcntEffs are identified segregating in the Extreme (43; 14.8%) and the Very_High (40; 13.7%) UVindex categories. Furthermore, the ORF1ab, which occupies two-thirds of the SARS-CoV-2 genome and expresses into 16 non-structural proteins (NSPs), harbors the highest number (163) of missense rcntEffs. We also observed that the nucleocapsid protein (N) and spike glycoprotein (S) encoding genes carry the second and third highest number of missense rcntEffs, 43 and 40, respectively. The rcntEffs counts observed in all UVindex categories are presented in Figure 5.

### Comparative genomic analysis

**Figure 6:**
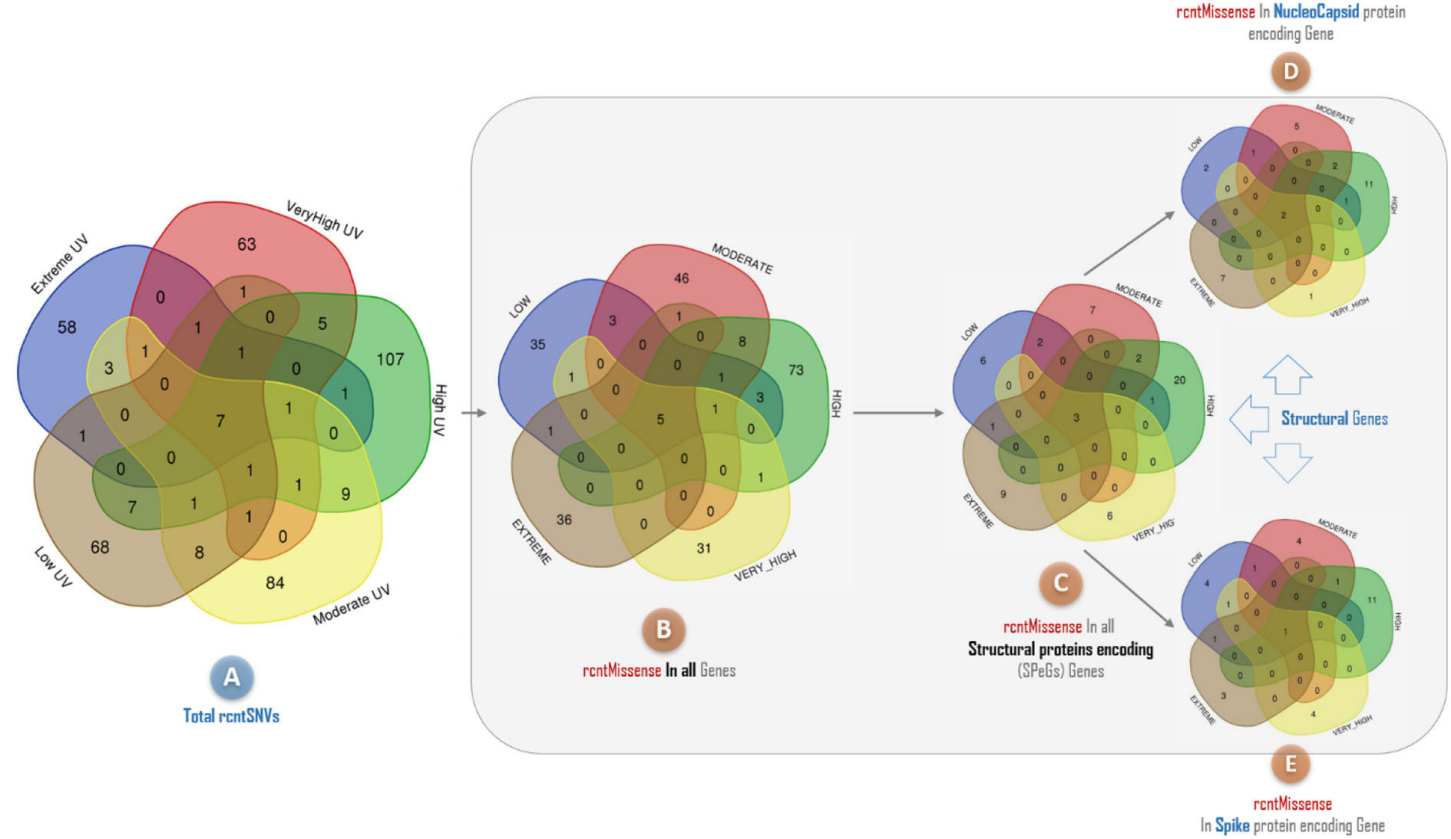
A Venn diagram depicting the overlap of recurrent single nucleotide variants (rcntSNVs) found across different SARS-CoV-2 populations from five WHO's defined UVindex country categories. The comparison based on total identified rcntSNVs across all 2,500 SARS-CoV-2 genomes from UVindex categories; Extreme (blue), Very_High (red), High (green), Moderate (yellow), and Low (brown) revealed a total of seven commonly shared variants **(A)**. The complete description of all UVindex categories is presented in the method section. Upon detailed functional annotation, all seven commonly shared rcntSNVs are found with five shared missense functional effects (rcntMissense-effects) on gene's functions **(B)**, of which three shared rcntMissense-effects are revealed in structural protein-encoding genes **(C)**, comprising two in nucleocapsid **(D)** and one in spike **(E)**. In all Venn diagrams, the UVindex-specific rcntSNVs/Missense-effects (CaSp-rcntSNVs/Effs) counts are given near the outer edges, whereas the shared rcntSNVs/effects are represented in the dark brown core middle of each diagram.

The rcntSNVs-based comparative analysis of all studied full-length SARS-CoV-2 genomes revealed a total of 380 (~73.8% of the all rcntSNVs) UVindex category-specific rcntSNVs (*CaSp*-rcntSNVs), not being shared among any two or more categories (Extreme 58, Very_High 63, High 107, Moderate 84, and Low 68). The comprehensive annotation of each category-specific rcntSNV is given in Supplementary_info_file.docx, Supplementary Tables 2–6. A total of seven rcntSNVs, five missense and two synonymous, observed commonly shared among all UVindex categories, with at least 3,217 overall recurrences, suggesting that all these common rcntSNVs are conserved and near to fixation (rcntSNVs-based comparison is shown in Figure 6A). Of seven shared rcntSNVs, the ORF1ab 14159C>T (missense; Pro4720Leu) is the most common rcntSNV found in RNA-dependent RNA polymerase (missense; RdRp Pro327Leu; 4,683/8,631 samples), followed by the N gene 608G>A (missense; N Arg203Lys; samples 35,98/8,631), 610G>C (missense; N Gly204Arg; 3,384/8,631 samples), S gene 1841A>G (missense; S Asp614Gly; samples 3,259/8,631), ORF1ab gene 2772C>T (synonymous; ORF1ab Phe924Phe; samples 3,238/8,631), and N gene 610G>C (synonymous; N Gly204Arg; samples 3,217/8,631). All commonly shared rcntSNVs and their respective annotations are given in Supplementary_info_file.docx, Supplementary Table 8. To effectively combat COVID-19, all seven commonly shared rcntSNVs may play a key role in universal vaccine preparation against SARS-CoV-2.

Functional annotation of all 380 *CaSp*-rcntSNVs revealed a sum of 420 category-specific rcntSNV effects (*CaSp*-rcntEffs) on genes products (Extreme 64, Very_High 68, High 120, Moderate 94, and Low 74). Of the total genes, the ORF1ab harbors the highest number of *CaSp*-rcntEffs (234), followed by all four structural genes (103) and six accessory genes (73). The detailed number of *CaSp*-rcntEffs loads per gene for each of the UVindex categories is given in Table 2.

**Table 2:** Functional effects of all identified category-specific recurrent SNVs (CaSp-rcntEffs) counts identified in all 2,500 SARS-CoV-2 genomes and their respective per WHO's defined UVindex category distribution.

**rcntEffs load on**	**Structural Genes**				**non-Structural Protein gene**	**Accessory Protein Genes**						**Total**
	N gene	S gene	M gene	E gene	ORF1ab	ORF3a	ORF10	ORF8	ORF7b	ORF7a	ORF6	
Extreme	07	06	03	01	38	01	02	03	00	01	02	64
Very_High	02	11	02	02	37	05	01	01	02	03	02	68
High	16	15	05	02	60	02	04	07	03	04	02	120
Moderate	11	07	03	02	54	06	03	05	02	00	01	94
Low	05	10	01	02	45	07	02	02	00	00	00	74
TOTAL rcntEffs	**41**	**49**	**14**	**09**	**234**	**21**	**12**	**18**	**07**	**08**	**7**	**420**

Of the total Uvindex *CaSp*-rcntEffs, 222 are found changing codons to specify biochemically different amino acids (*CaSp*-rcntMissense-effects), 136 are observed without consequent changes in the amino-acid compositions (*CaSp*-rcntSilent-effects), and 62 are detected in the genes' regulatory region (*CaSp*-rcntRegulatory-effects). Most *CaSp*-rcntMissense effects observed in ORF1ab (141), S (27), and surprisingly, the N (26) protein-encoding structural genes. These results showed that SARS-CoV-2 capitalized *CaSp*-rcntMissense, likely the gain of function variant, in ORF1ab and structural protein-encoding genes to adapt to varying UVindex ranges (Table 3).

**Table 3:** Functional effects of all identified category-specific recurrent SNVs (CaSp-rcntEffs) count across all SARS-CoV-2 genes.

**Genes groups**	**Genes**	Missense ^*^[Ex:Vh:Hi:Mo:Lo]	**Synonymous [Silent]**	**Regulatory**	**Total**	**Effects per gene group**
Non-structural genes	ORF1ab	141 [22:21:42:32:24]	85	08	234	**234**
Structural genes	E Protein	03 [0:1:1:0:1]	01	05	09	
	M Protein	02 [1:1:0:0:0]	08	04	14	
	N Protein	26 [7:1:11:5:2]	08	07	41	**113**
	S Protein	27 [3:5:11:4:4]	20	02	49	
Accessory genes	ORF3a	09 [1:1:1:2:4]	07	05	21	
	ORF6	03 [1:0:1:1:0]	00	04	07	
	ORF7a	03 [0:2:1:0:0]	02	03	08	**73**
	ORF7b	00 [0:0:0:0:0]	01	06	07	
	ORF8	07 [1:0:4:2:0]	04	07	18	
	ORF10	01 [0:0:1:0:0]	00	11	12	
**Grand total**		**222**	**136**	**62**	**420**	

The horizontal-rows and vertical columns represent the CaSp-rcntEffs count by functional effect classes and by gene groups, respectively.

* [Ex:Vh:Hi:Mo:Lo] denotes the UVindex-specific rcntSNV-missense effect counts in Extreme, Very_High, High, Moderate, and Low categories, respectively.

**Figure 7:**
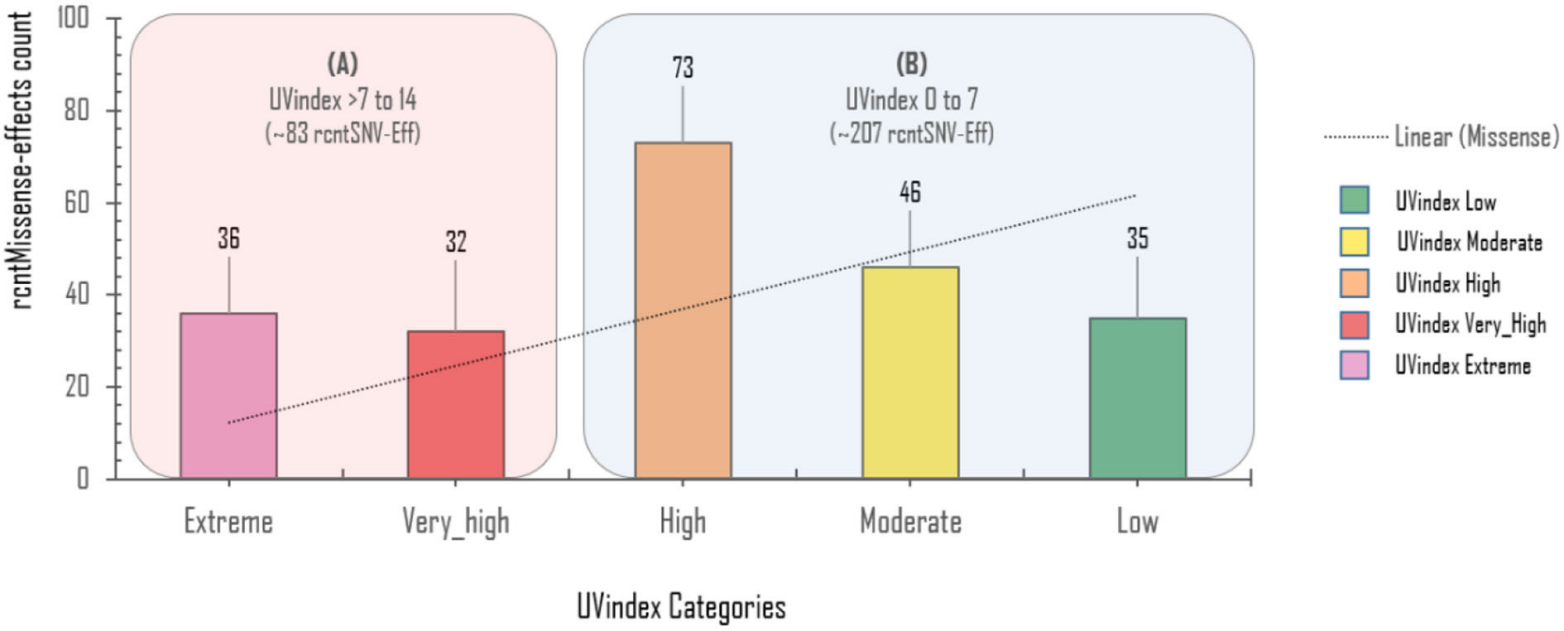
Per UVindex category-specific rcntMissense-effects (CaSp-rcntMissense) count. The bars from left to right shows the total identified number of CaSp-rcntMissense effects in extreme (43; 14.8%), very high (40; 13.7%), high (92; 31.7%), moderate (65; 22.4%), and low (50; 17.2%) UVindex categories. The plot **(A)** reveals countries with UVindex above seven impose strong negative selection pressure by allowing least number of SARS-CoV-2 variants with minimal identified CaSp-rcntMissense effects (~28.62%), whereas, **(B)** most number of CaSp-rcntEffs (~71.38%) are observed in group of countries experiencing UVindex from 0 to 7.

Approximately 69.4% (154/222) of the overall *CaSp*-rcntMissense effects are detected in the UVindex range ≤ 7 (UVindex categories: Low 35, Moderate 46, and High 73), whereas the remaining 30.6% (68/222) are observed in the Extreme UVindex (36) and Very_High UVindex (31) categories (for details, see Figures 6B–E). The negatively related linear-trending line with the UVindex implies that the UVindex is inversely proportional to the *CaSp*-rcntMissense effects count. Suggesting that a higher UVindex (mostly ≥ 8) allows significantly fewer SARS-CoV-2 viral strains to survive hence imposing strong negative selection pressure (Figure 7). A set of all category-specific rcntMissense effects causing rcntSNVs may serve as potential resource for considerably more effective region-specific vaccine production.

### Phylogeny

**Figure 8:**
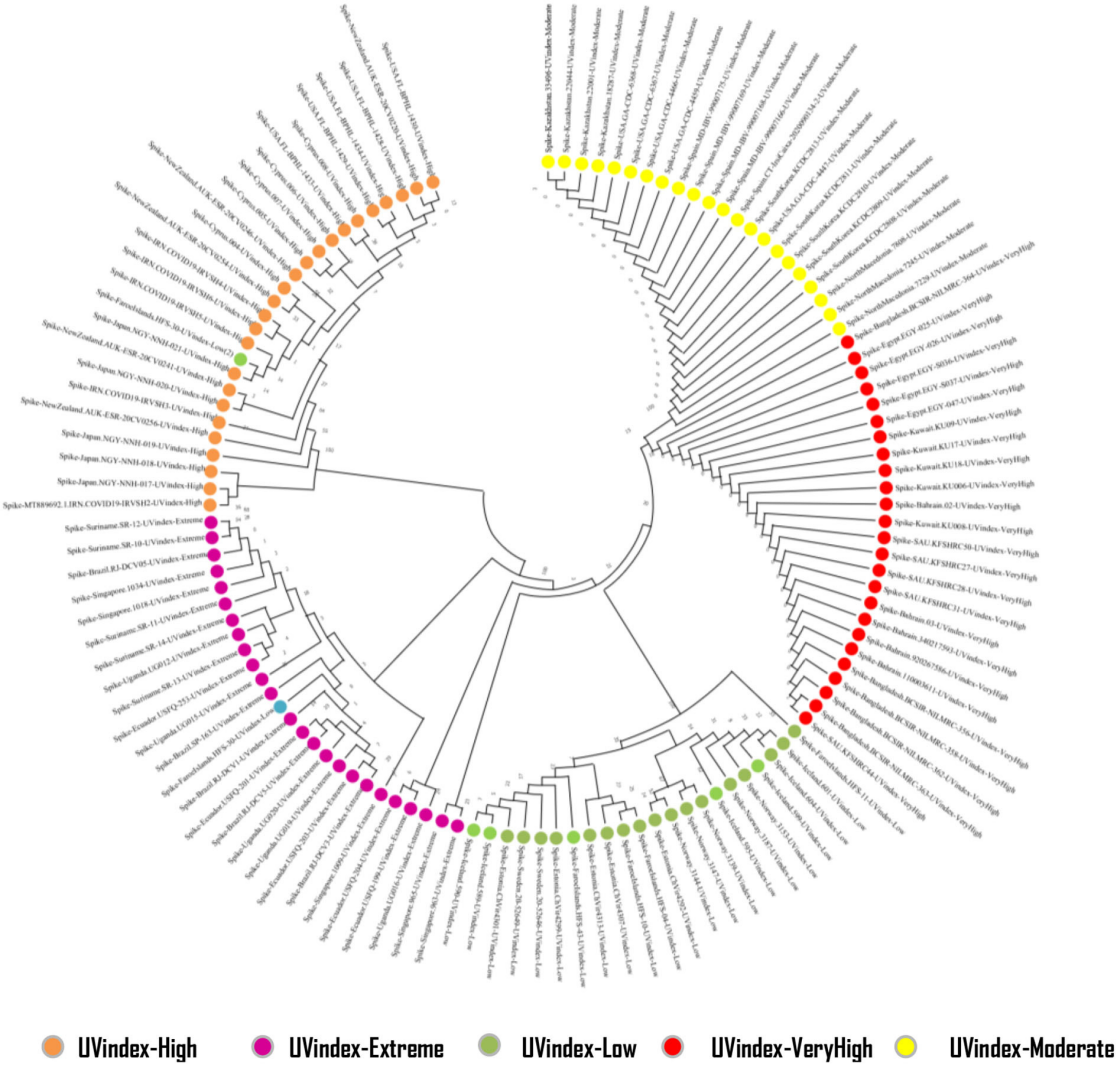
Phylogenetic tree of SARS-CoV-2 genome sequences prevalent in five different UVindex regions. The five beta corona viral populations constituted five different clades. The SARS-CoV-2 population from high UVindex regions was found as the outgroup clade, whereas the SARS-CoV-2 populations from extreme, low, very high, and high UVindex regions formed three descendant clades within the ingroup.

To find the evolutionary relationship between SARS-CoV-2 populations prevailing in different UVindex regions, we constructed a phylogenetic tree based on high-quality whole-genome sequences of 125 randomly selected SARS-CoV-2 samples, 25 from each of the UVindex categories (Figure 8).

Our phylogenetic analysis revealed five different branches for all randomly selected 125 high-quality SARS-CoV-2 genome samples (25 from each of the UVindex region). The tree displays separate branches for SARS-CoV-2 retrieved from UVindex regions, namely High (orange), Extreme (purple), Low (green), Very_High (red), and Moderate (yellow). The phylogenetic analysis has shown High UVindex inhabiting SARS-CoV-2 population as an outgroup and the SARS-CoV-2 prevailing Extreme, Low, Very_High, and Moderate UVindex regions as ingroup populations. To accommodate four SARS-CoV-2 populations, three main lineages were found within the ingroup, revealing the extent of relationships between different populations. The Extreme and Low UVindex populations are placed in two separate ingroup lineages and SARS-CoV-2 populations from the Very_High and Moderate UVindex regions are found sharing the third lineage. This relationship reflects that all SARS-CoV-2 samples, which are included in this study, are descended from the High UVindex region's inhabiting populations, whereas the SARS-CoV-2 populations from Very_High and Moderate UVindex regions are closely related to others.

## Conclusion

SARS-CoV-2 is the pandemic COVID-19-causing coronavirus, which has raised a great threat to human health in almost all regions of the world. The genome-wide analysis of the rapidly evolving SARS-CoV-2 genomes discovered a large majority of the rcntSNVs as distinctive (found uniquely in a specific UVindex region), revealing the SARS-CoV-2 differential genomic responses to WHO's defined five different UVindex regions. Based on the total number of rcntSNVs predicted in all included SARS-CoV-2 genomes, our analysis showed that the Extreme UVindex applies negative selection pressure, whereas UVindex range of 6–7 provides the most suitable conditions for SARS-CoV-2 endurance. The phylogenetic relationship indicated the high UVindex region inhabiting the SARS-CoV-2 population as the recent progenitor of all included samples. To help in immune evasion and tolerate the DNA damaging effects of varying UV-solar radiation, the SARS-CoV-2 has acquired the highest number of missense rcntSNVs in their spike glycoprotein and nucleocapsid-encoding genes. Since COVID-19 diagnostic tests and vaccines are based on the spike or the nucleocapsid viral proteins, all missense rcntSNVs may need to be included in future diagnostic and vaccine formulations.

## Data availability statement

The original contributions presented in the study are included in the article/Supplementary material, further inquiries can be directed to the corresponding author.

## Author contributions

Writing—review and editing: MR, MA, AF, SM, NA, XL, FN, and NI. Writing—original draft preparation: NI, M, AF, and SK. Validation: NI, TY, RR, and XL. Supervision and project administration: SM. Software: NI, TY, and SK. Resources: NI, SM, MJ, and XL. Methodology: NI, TY, XL, and MR. Investigation: NI, MR, MA, and FN. Funding acquisition, conceptualization, and formal analysis: NI. Data curation: NI, M, MJ, SK, and ST. All authors contributed to the article and approved the submitted version.

## Conflict of interest

The authors declare that the research was conducted in the absence of any commercial or financial relationships that could be construed as a potential conflict of interest.

## Publisher's note

All claims expressed in this article are solely those of the authors and do not necessarily represent those of their affiliated organizations, or those of the publisher, the editors and the reviewers. Any product that may be evaluated in this article, or claim that may be made by its manufacturer, is not guaranteed or endorsed by the publisher.
