# Supplementary material for: The SARS-CoV-2 differential genomic adaptation in response to varying UVindex reveals potential genomic resources for better COVID-19 diagnosis and prevention
Source: Front Microbiol. 2022 Aug 4;13:922393. doi: 10.3389/fmicb.2022.922393 (PMC9396647; doi:10.3389/fmicb.2022.922393)
Supplement: Supplementary file 1 [file Data_Sheet_1.docx]

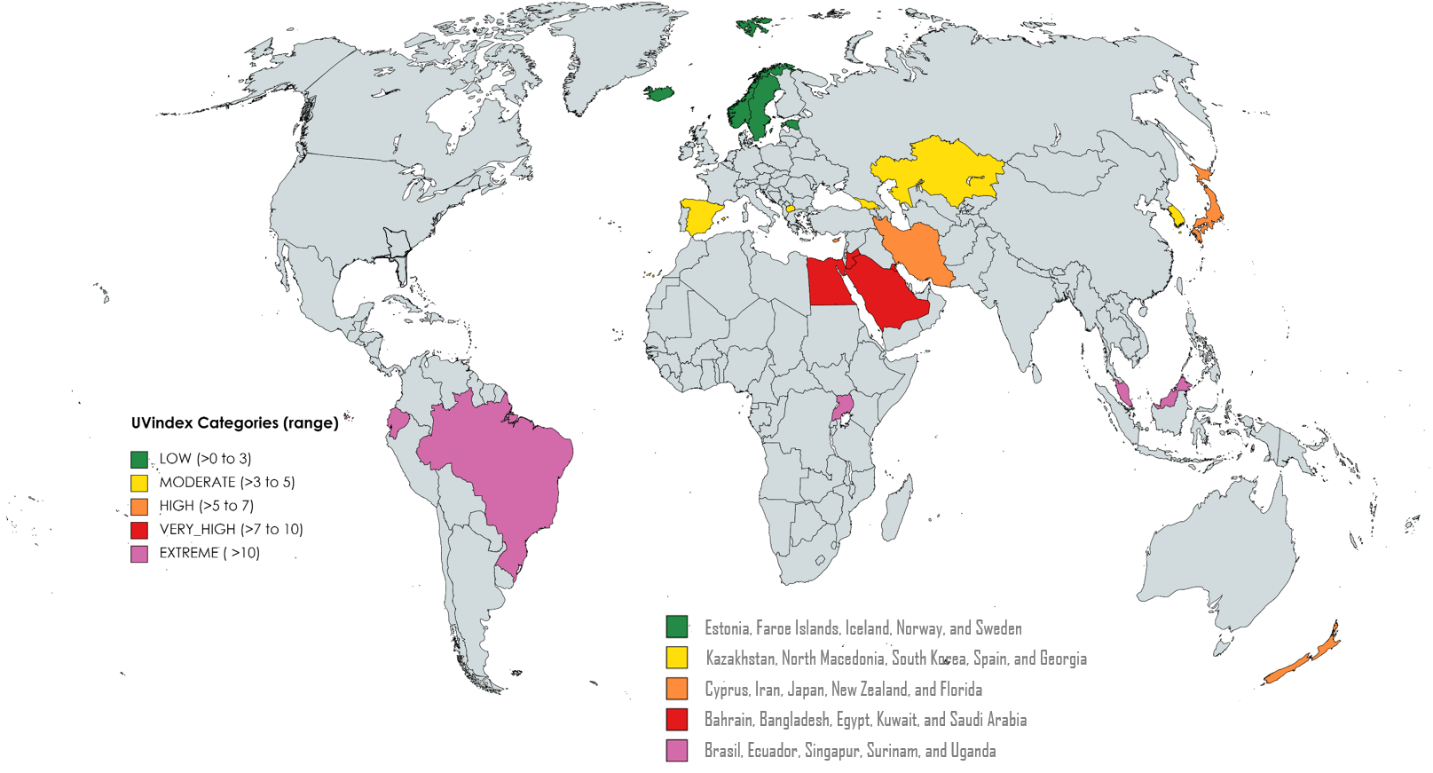


Supplementary Map 1: Map of all included countries. In each of the WHO defined UVindex country’s category, five countries were included based on the highest recorded UVindex ranges. The color highlighted countries are the selected countries in this study and following the WHO’s UVindex color codes, each included country is assigned a color according to UVindex categories.

Supplementary Table 1: Top five countries in their respective UVindex categories.

| **LOW** | **MODERATE** | **HIGH** | **VERY_HIGH** | **EXTREME** |
| --- | --- | --- | --- | --- |
| Estonia | Kazakhstan | Cyprus | Bahrain | Brazil |
| Faroe Islands | North Macedonia | Iran | Bangladesh | Ecuador |
| Iceland | South Korea | Japan | Egypt | Singapore |
| Norway | Spain | New Zealand | Kuwait | Suriname |
| Sweden | Georgia | Florida | Saudi Arabia SAU | Uganda |

Supplementary Table 2: Low UVindex Specific rcntSNVs

| **rcntSNVs** | **Gene** | **Functional_rcntEffs** | **A.Acid-Change** | **rcntSNV_Impact** |
| --- | --- | --- | --- | --- |
| n.44C>T | CHR_START-ORF1ab | intergenic_region |  | MODIFIER |
| n.106C>T | CHR_START-ORF1ab | intergenic_region |  | MODIFIER |
| c.-44C>T | ORF1ab | upstream_gene_variant |  | MODIFIER |
| c.70C>T | ORF1ab | missense_variant | p.Arg24Cys | MODERATE |
| c.101C>T | ORF1ab | missense_variant | p.Ser34Phe | MODERATE |
| c.507T>C | ORF1ab | synonymous_variant | p.Val169Val | LOW |
| c.953C>T | ORF1ab | missense_variant | p.Ser318Leu | MODERATE |
| c.998C>T | ORF1ab | missense_variant | p.Thr333Met | MODERATE |
| c.1037C>T | ORF1ab | missense_variant | p.Thr346Ile | MODERATE |
| c.1293A>G | ORF1ab | missense_variant | p.Ile431Met | MODERATE |
| c.1329C>T | ORF1ab | synonymous_variant | p.Ser443Ser | LOW |
| c.1703C>T | ORF1ab | missense_variant | p.Thr568Ile | MODERATE |
| c.3330T>C | ORF1ab | synonymous_variant | p.Leu1110Leu | LOW |
| c.3421C>T | ORF1ab | missense_variant | p.His1141Tyr | MODERATE |
| c.3808C>T | ORF1ab | missense_variant | p.Leu1270Phe | MODERATE |
| c.3819C>T | ORF1ab | synonymous_variant | p.Asp1273Asp | LOW |
| c.3969C>T | ORF1ab | synonymous_variant | p.Asp1323Asp | LOW |
| c.4877C>T | ORF1ab | missense_variant | p.Thr1626Ile | MODERATE |
| c.4905C>T | ORF1ab | synonymous_variant | p.Tyr1635Tyr | LOW |
| c.5931C>T | ORF1ab | synonymous_variant | p.Pro1977Pro | LOW |
| c.6020C>T | ORF1ab | missense_variant | p.Thr2007Ile | MODERATE |
| c.6135A>G | ORF1ab | synonymous_variant | p.Lys2045Lys | LOW |
| c.6180C>T | ORF1ab | synonymous_variant | p.Asp2060Asp | LOW |
| c.6271G>A | ORF1ab | missense_variant | p.Gly2091Ser | MODERATE |
| c.6489T>C | ORF1ab | synonymous_variant | p.Arg2163Arg | LOW |
| c.7569C>T | ORF1ab | synonymous_variant | p.Asn2523Asn | LOW |
| c.7872G>T | ORF1ab | synonymous_variant | p.Val2624Val | LOW |
| c.8403C>T | ORF1ab | synonymous_variant | p.Asp2801Asp | LOW |
| c.10014C>T | ORF1ab | synonymous_variant | p.Leu3338Leu | LOW |
| c.10058A>G | ORF1ab | missense_variant | p.Lys3353Arg | MODERATE |
| c.10581G>T | ORF1ab | missense_variant | p.Met3527Ile | MODERATE |
| c.10603C>T | ORF1ab | synonymous_variant | p.Leu3535Leu | LOW |
| c.10867G>T | ORF1ab | missense_variant | p.Ala3623Ser | MODERATE |
| c.11385C>T | ORF1ab | synonymous_variant | p.Gly3795Gly | LOW |
| c.11517A>G | ORF1ab | synonymous_variant | p.Lys3839Lys | LOW |
| c.14889G>T | ORF1ab | missense_variant | p.Met4963Ile | MODERATE |
| c.15403G>T | ORF1ab | missense_variant | p.Asp5135Tyr | MODERATE |
| c.15826G>T | ORF1ab | missense_variant | p.Asp5276Tyr | MODERATE |
| c.15868G>T | ORF1ab | missense_variant | p.Asp5290Tyr | MODERATE |
| c.16557C>T | ORF1ab | synonymous_variant | p.Asn5519Asn | LOW |
| c.16998T>C | ORF1ab | synonymous_variant | p.Arg5666Arg | LOW |
| c.18006G>T | ORF1ab | missense_variant | p.Met6002Ile | MODERATE |
| c.18321C>T | ORF1ab | synonymous_variant | p.Leu6107Leu | LOW |
| c.18896C>T | ORF1ab | missense_variant | p.Ser6299Phe | MODERATE |
| c.20398C>T | ORF1ab | missense_variant | p.Pro6800Ser | MODERATE |
| c.21122A>G | ORF1ab | missense_variant | p.Gln7041Arg | MODERATE |
| c.21205C>A | ORF1ab | missense_variant | p.Gln7069Lys | MODERATE |
| c.177T>C | S | synonymous_variant | p.Phe59Phe | LOW |
| c.1067A>G | S | missense_variant | p.Lys356Arg | MODERATE |
| c.1107T>C | S | synonymous_variant | p.Tyr369Tyr | LOW |
| c.1876G>T | S | missense_variant | p.Ala626Ser | MODERATE |
| c.2198A>G | S | missense_variant | p.Lys733Arg | MODERATE |
| c.2289A>G | S | synonymous_variant | p.Leu763Leu | LOW |
| c.3711G>C | S | missense_variant | p.Met1237Ile | MODERATE |
| c.3738C>T | S | synonymous_variant | p.Gly1246Gly | LOW |
| c.100A>G | ORF3a | missense_variant | p.Thr34Ala | MODERATE |
| c.472A>G | ORF3a | missense_variant | p.Ile158Val | MODERATE |
| c.530G>T | ORF3a | missense_variant | p.Ser177Ile | MODERATE |
| c.618C>T | ORF3a | synonymous_variant | p.Tyr206Tyr | LOW |
| c.752G>T | ORF3a | missense_variant | p.Gly251Val | MODERATE |
| c.203C>T | E | missense_variant | p.Ser68Phe | MODERATE |
| c.189T>C | ORF8 | synonymous_variant | p.Asp63Asp | LOW |
| c.60C>T | N | synonymous_variant | p.Pro20Pro | LOW |
| c.539G>T | N | missense_variant | p.Ser180Ile | MODERATE |
| c.632C>T | N | missense_variant | p.Ala211Val | MODERATE |
| c.801A>G | N | synonymous_variant | p.Ala267Ala | LOW |
| c.-13C>T | ORF10 | upstream_gene_variant |  | MODIFIER |
| c.*32G>A | ORF10 | downstream_gene_variant |  | MODIFIER |

Supplementary Table 3: Moderate UVindex Specific rcntSNVs

| **rcntSNVs** | **Gene** | **Functional_rcntEffs** | **A.Acid-Change** | **rcntSNV_Impact** |
| --- | --- | --- | --- | --- |
| c.-62G>T | ORF1ab | upstream_gene_variant |  | MODIFIER |
| c.-25C>A | ORF1ab | upstream_gene_variant |  | MODIFIER |
| c.617C>T | ORF1ab | missense_variant | p.Ala206Val | MODERATE |
| c.667G>T | ORF1ab | missense_variant | p.Asp223Tyr | MODERATE |
| c.925C>T | ORF1ab | missense_variant | p.Pro309Ser | MODERATE |
| c.1673C>T | ORF1ab | missense_variant | p.Ser558Phe | MODERATE |
| c.1722A>G | ORF1ab | synonymous_variant | p.Ser574Ser | LOW |
| c.2021G>A | ORF1ab | missense_variant | p.Ser674Asn | MODERATE |
| c.2792A>G | ORF1ab | missense_variant | p.Glu931Gly | MODERATE |
| c.3354C>T | ORF1ab | synonymous_variant | p.Val1118Val | LOW |
| c.3932A>G | ORF1ab | missense_variant | p.Glu1311Gly | MODERATE |
| c.3990G>A | ORF1ab | synonymous_variant | p.Pro1330Pro | LOW |
| c.4611C>T | ORF1ab | synonymous_variant | p.Tyr1537Tyr | LOW |
| c.5280A>T | ORF1ab | synonymous_variant | p.Thr1760Thr | LOW |
| c.5519C>T | ORF1ab | missense_variant | p.Thr1840Ile | MODERATE |
| c.6264A>G | ORF1ab | synonymous_variant | p.Glu2088Glu | LOW |
| c.6386C>T | ORF1ab | missense_variant | p.Ala2129Val | MODERATE |
| c.6725C>T | ORF1ab | missense_variant | p.Ser2242Phe | MODERATE |
| c.7818G>A | ORF1ab | missense_variant | p.Met2606Ile | MODERATE |
| c.8087C>T | ORF1ab | missense_variant | p.Ala2696Val | MODERATE |
| c.9212T>A | ORF1ab | missense_variant | p.Phe3071Tyr | MODERATE |
| c.9261G>T | ORF1ab | missense_variant | p.Met3087Ile | MODERATE |
| c.9545C>T | ORF1ab | missense_variant | p.Thr3182Ile | MODERATE |
| c.9583A>G | ORF1ab | missense_variant | p.Ser3195Gly | MODERATE |
| c.9702C>T | ORF1ab | synonymous_variant | p.Leu3234Leu | LOW |
| c.9774C>T | ORF1ab | synonymous_variant | p.Thr3258Thr | LOW |
| c.10000G>A | ORF1ab | missense_variant | p.Gly3334Ser | MODERATE |
| c.10191C>T | ORF1ab | synonymous_variant | p.Phe3397Phe | LOW |
| c.10482C>T | ORF1ab | synonymous_variant | p.Asn3494Asn | LOW |
| c.10490C>T | ORF1ab | missense_variant | p.Ala3497Val | MODERATE |
| c.11265C>T | ORF1ab | synonymous_variant | p.Ala3755Ala | LOW |
| c.11630A>T | ORF1ab | missense_variant | p.Gln3877Leu | MODERATE |
| c.11651C>T | ORF1ab | missense_variant | p.Ser3884Leu | MODERATE |
| c.12489C>T | ORF1ab | synonymous_variant | p.Cys4163Cys | LOW |
| c.12651A>T | ORF1ab | synonymous_variant | p.Thr4217Thr | LOW |
| c.13008T>C | ORF1ab | synonymous_variant | p.His4336His | LOW |
| c.15103C>T | ORF1ab | missense_variant | p.Leu5035Phe | MODERATE |
| c.15195G>T | ORF1ab | missense_variant | p.Met5065Ile | MODERATE |
| c.15723A>G | ORF1ab | synonymous_variant | p.Val5241Val | LOW |
| c.16401C>T | ORF1ab | synonymous_variant | p.Leu5467Leu | LOW |
| c.17563T>C | ORF1ab | synonymous_variant | p.Leu5855Leu | LOW |
| c.18271G>A | ORF1ab | missense_variant | p.Val6091Ile | MODERATE |
| c.18636C>T | ORF1ab | synonymous_variant | p.Val6212Val | LOW |
| c.18749C>T | ORF1ab | missense_variant | p.Ala6250Val | MODERATE |
| c.19024C>A | ORF1ab | missense_variant | p.Pro6342Thr | MODERATE |
| c.19091G>T | ORF1ab | missense_variant | p.Ser6364Ile | MODERATE |
| c.19469C>T | ORF1ab | missense_variant | p.Thr6490Ile | MODERATE |
| c.19486G>T | ORF1ab | missense_variant | p.Asp6496Tyr | MODERATE |
| c.19672G>A | ORF1ab | missense_variant | p.Val6558Ile | MODERATE |
| c.19713G>T | ORF1ab | synonymous_variant | p.Thr6571Thr | LOW |
| c.20021C>T | ORF1ab | missense_variant | p.Ala6674Val | MODERATE |
| c.20380C>T | ORF1ab | missense_variant | p.His6794Tyr | MODERATE |
| c.20426A>T | ORF1ab | missense_variant | p.Gln6809Leu | MODERATE |
| c.459G>T | S | missense_variant | p.Met153Ile | MODERATE |
| c.530T>C | S | missense_variant | p.Met177Thr | MODERATE |
| c.641G>T | S | missense_variant | p.Arg214Leu | MODERATE |
| c.1032C>G | S | synonymous_variant | p.Ala344Ala | LOW |
| c.1569T>C | S | synonymous_variant | p.Thr523Thr | LOW |
| c.1869T>C | S | synonymous_variant | p.Ala623Ala | LOW |
| c.2074A>T | S | missense_variant | p.Ile692Phe | MODERATE |
| c.318C>T | ORF3a | synonymous_variant | p.Leu106Leu | LOW |
| c.327T>C | ORF3a | synonymous_variant | p.Tyr109Tyr | LOW |
| c.467A>G | ORF3a | missense_variant | p.Tyr156Cys | MODERATE |
| c.587G>T | ORF3a | missense_variant | p.Gly196Val | MODERATE |
| c.738T>C | ORF3a | synonymous_variant | p.Ile246Ile | LOW |
| c.12C>T | E | synonymous_variant | p.Phe4Phe | LOW |
| c.87T>C | M | synonymous_variant | p.Leu29Leu | LOW |
| c.306G>A | M | synonymous_variant | p.Leu102Leu | LOW |
| c.23A>G | ORF6 | missense_variant | p.Gln8Arg | MODERATE |
| c.22G>A | ORF8 | missense_variant | p.Gly8Arg | MODERATE |
| c.51C>T | ORF8 | synonymous_variant | p.His17His | LOW |
| c.260C>T | ORF8 | missense_variant | p.Thr87Ile | MODERATE |
| c.27G>C | N | missense_variant | p.Gln9His | MODERATE |
| c.103G>T | N | missense_variant | p.Ala35Ser | MODERATE |
| c.384C>T | N | synonymous_variant | p.Asp128Asp | LOW |
| c.442A>T | N | missense_variant | p.Thr148Ser | MODERATE |
| c.486T>C | N | synonymous_variant | p.Pro162Pro | LOW |
| c.590C>T | N | missense_variant | p.Ser197Leu | MODERATE |
| c.617C>T | N | missense_variant | p.Ser206Phe | MODERATE |
| c.804C>T | N | synonymous_variant | p.Tyr268Tyr | LOW |
| c.906G>T | N | synonymous_variant | p.Pro302Pro | LOW |
| c.-18G>A | ORF10 | upstream_gene_variant |  | MODIFIER |
| c.*36T>A | ORF10 | downstream_gene_variant |  | MODIFIER |
| c.*105G>T | ORF10 | downstream_gene_variant |  | MODIFIER |

Supplementary Table 4: High UVindex Specific rcntSNVs

| **rcntSNVs** | **Gene** | **Functional_rcntEffs** | **AminoAcid-Change** | **rcntSNV_Impact** |
| --- | --- | --- | --- | --- |
| n.21C>T | CHR_START-ORF1ab | intergenic_region |  | MODIFIER |
| c.-79A>G | ORF1ab | upstream_gene_variant |  | MODIFIER |
| c.-8A>C | ORF1ab | upstream_gene_variant |  | MODIFIER |
| c.404G>A | ORF1ab | missense_variant | p.Ser135Asn | MODERATE |
| c.482G>T | ORF1ab | missense_variant | p.Trp161Leu | MODERATE |
| c.936C>T | ORF1ab | synonymous_variant | p.Cys312Cys | LOW |
| c.947G>C | ORF1ab | missense_variant | p.Cys316Ser | MODERATE |
| c.1466A>C | ORF1ab | missense_variant | p.Glu489Ala | MODERATE |
| c.1520G>C | ORF1ab | missense_variant | p.Gly507Ala | MODERATE |
| c.1696G>A | ORF1ab | missense_variant | p.Ala566Thr | MODERATE |
| c.2119G>C | ORF1ab | missense_variant | p.Glu707Gln | MODERATE |
| c.2419A>C | ORF1ab | missense_variant | p.Met807Leu | MODERATE |
| c.2550T>C | ORF1ab | synonymous_variant | p.Asp850Asp | LOW |
| c.2860G>C | ORF1ab | missense_variant | p.Asp954His | MODERATE |
| c.3104C>T | ORF1ab | missense_variant | p.Thr1035Ile | MODERATE |
| c.3140A>C | ORF1ab | missense_variant | p.Glu1047Ala | MODERATE |
| c.3142G>C | ORF1ab | missense_variant | p.Glu1048Gln | MODERATE |
| c.3146C>T | ORF1ab | missense_variant | p.Ala1049Val | MODERATE |
| c.3149A>C | ORF1ab | missense_variant | p.Lys1050Thr | MODERATE |
| c.3216A>C | ORF1ab | synonymous_variant | p.Ala1072Ala | LOW |
| c.3340T>C | ORF1ab | missense_variant | p.Cys1114Arg | MODERATE |
| c.3369C>T | ORF1ab | synonymous_variant | p.Asn1123Asn | LOW |
| c.3737C>T | ORF1ab | missense_variant | p.Thr1246Ile | MODERATE |
| c.4081T>C | ORF1ab | missense_variant | p.Ser1361Pro | MODERATE |
| c.4331C>T | ORF1ab | missense_variant | p.Thr1444Ile | MODERATE |
| c.5318C>T | ORF1ab | missense_variant | p.Thr1773Ile | MODERATE |
| c.6263A>G | ORF1ab | missense_variant | p.Glu2088Gly | MODERATE |
| c.6348A>G | ORF1ab | synonymous_variant | p.Val2116Val | LOW |
| c.6412A>C | ORF1ab | missense_variant | p.Ile2138Leu | MODERATE |
| c.6476G>T | ORF1ab | missense_variant | p.Arg2159Leu | MODERATE |
| c.7128G>T | ORF1ab | synonymous_variant | p.Pro2376Pro | LOW |
| c.7752G>T | ORF1ab | synonymous_variant | p.Ala2584Ala | LOW |
| c.7787A>G | ORF1ab | missense_variant | p.Asn2596Ser | MODERATE |
| c.7922T>C | ORF1ab | missense_variant | p.Phe2641Ser | MODERATE |
| c.7939G>A | ORF1ab | missense_variant | p.Glu2647Lys | MODERATE |
| c.8442T>C | ORF1ab | synonymous_variant | p.Gly2814Gly | LOW |
| c.8807C>A | ORF1ab | missense_variant | p.Thr2936Asn | MODERATE |
| c.8987T>C | ORF1ab | missense_variant | p.Val2996Ala | MODERATE |
| c.9021C>T | ORF1ab | synonymous_variant | p.Asn3007Asn | LOW |
| c.9095C>T | ORF1ab | missense_variant | p.Thr3032Ile | MODERATE |
| c.9140T>C | ORF1ab | missense_variant | p.Ile3047Thr | MODERATE |
| c.9832G>A | ORF1ab | missense_variant | p.Gly3278Ser | MODERATE |
| c.10111C>T | ORF1ab | missense_variant | p.Pro3371Ser | MODERATE |
| c.10439G>A | ORF1ab | missense_variant | p.Arg3480Lys | MODERATE |
| c.10897C>A | ORF1ab | missense_variant | p.His3633Asn | MODERATE |
| c.11360G>A | ORF1ab | missense_variant | p.Gly3787Asp | MODERATE |
| c.11400C>T | ORF1ab | synonymous_variant | p.Leu3800Leu | LOW |
| c.11960C>T | ORF1ab | missense_variant | p.Ala3987Val | MODERATE |
| c.13743A>G | ORF1ab | synonymous_variant | p.Gln4581Gln | LOW |
| c.14459C>T | ORF1ab | missense_variant | p.Ala4820Val | MODERATE |
| c.15618T>G | ORF1ab | synonymous_variant | p.Pro5206Pro | LOW |
| c.15737A>G | ORF1ab | missense_variant | p.Lys5246Arg | MODERATE |
| c.15795A>T | ORF1ab | synonymous_variant | p.Pro5265Pro | LOW |
| c.16011C>T | ORF1ab | synonymous_variant | p.Cys5337Cys | LOW |
| c.16367C>T | ORF1ab | missense_variant | p.Thr5456Ile | MODERATE |
| c.18066G>A | ORF1ab | synonymous_variant | p.Glu6022Glu | LOW |
| c.18128C>T | ORF1ab | missense_variant | p.Thr6043Ile | MODERATE |
| c.19718G>A | ORF1ab | missense_variant | p.Cys6573Tyr | MODERATE |
| c.20638G>A | ORF1ab | missense_variant | p.Gly6880Arg | MODERATE |
| c.21128C>A | ORF1ab | missense_variant | p.Ser7043Tyr | MODERATE |
| c.65C>T | S | missense_variant | p.Thr22Ile | MODERATE |
| c.437A>G | S | missense_variant | p.His146Arg | MODERATE |
| c.458T>C | S | missense_variant | p.Met153Thr | MODERATE |
| c.595G>C | S | missense_variant | p.Gly199Arg | MODERATE |
| c.1032C>A | S | synonymous_variant | p.Ala344Ala | LOW |
| c.1151C>T | S | missense_variant | p.Pro384Leu | MODERATE |
| c.1173C>T | S | synonymous_variant | p.Cys391Cys | LOW |
| c.1462T>C | S | missense_variant | p.Cys488Arg | MODERATE |
| c.2169C>T | S | synonymous_variant | p.Thr723Thr | LOW |
| c.2423A>G | S | missense_variant | p.Asp808Gly | MODERATE |
| c.2514T>C | S | synonymous_variant | p.Gly838Gly | LOW |
| c.2842C>A | S | missense_variant | p.Leu948Ile | MODERATE |
| c.3208G>T | S | missense_variant | p.Ala1070Ser | MODERATE |
| c.3307T>C | S | missense_variant | p.Phe1103Leu | MODERATE |
| c.3575A>G | S | missense_variant | p.Asn1192Ser | MODERATE |
| c.293C>T | ORF3a | missense_variant | p.Ala98Val | MODERATE |
| c.83T>C | E | missense_variant | p.Leu28Pro | MODERATE |
| c.45G>A | M | synonymous_variant | p.Lys15Lys | LOW |
| c.102T>C | M | synonymous_variant | p.Leu34Leu | LOW |
| c.363C>T | M | synonymous_variant | p.Asn121Asn | LOW |
| c.504C>A | M | synonymous_variant | p.Ile168Ile | LOW |
| c.663A>G | M | synonymous_variant | p.Val221Val | LOW |
| c.145T>C | ORF6 | missense_variant | p.Tyr49His | MODERATE |
| c.194T>A | ORF7a | missense_variant | p.Phe65Tyr | MODERATE |
| c.210C>T | ORF7a | synonymous_variant | p.Gly70Gly | LOW |
| c.12T>A | ORF7b | synonymous_variant | p.Leu4Leu | LOW |
| c.11T>C | ORF8 | missense_variant | p.Leu4Pro | MODERATE |
| c.51C>G | ORF8 | missense_variant | p.His17Gln | MODERATE |
| c.241G>A | ORF8 | missense_variant | p.Val81Ile | MODERATE |
| c.327A>T | ORF8 | missense_variant | p.Leu109Phe | MODERATE |
| c.351T>C | ORF8 | synonymous_variant | p.Val117Val | LOW |
| c.17C>T | N | missense_variant | p.Pro6Leu | MODERATE |
| c.184G>C | N | missense_variant | p.Glu62Gln | MODERATE |
| c.452C>T | N | missense_variant | p.Pro151Leu | MODERATE |
| c.548C>A | N | missense_variant | p.Ser183Tyr | MODERATE |
| c.557C>T | N | missense_variant | p.Ser186Phe | MODERATE |
| c.581C>T | N | missense_variant | p.Ser194Leu | MODERATE |
| c.604A>T | N | missense_variant | p.Ser202Cys | MODERATE |
| c.605G>C | N | missense_variant | p.Ser202Thr | MODERATE |
| c.643G>C | N | missense_variant | p.Gly215Arg | MODERATE |
| c.874A>G | N | missense_variant | p.Ile292Val | MODERATE |
| c.1042G>A | N | missense_variant | p.Asp348Asn | MODERATE |
| c.1101G>A | N | synonymous_variant | p.Glu367Glu | LOW |
| c.-21C>A | ORF10 | upstream_gene_variant |  | MODIFIER |
| c.40T>C | ORF10 | missense_variant | p.Tyr14His | MODERATE |
| c.*7T>C | ORF10 | downstream_gene_variant |  | MODIFIER |
| c.*18G>T | ORF10 | downstream_gene_variant |  | MODIFIER |

Supplementary Table 5: Very_High UVindex Specific rcntSNVs

| **rcntSNVs** | **Gene** | **Functional_rcntEffs** | **A.Acid-Change** | **rcntSNV_Impact** |
| --- | --- | --- | --- | --- |
| c.-47G>T | ORF1ab | upstream_gene_variant |  | MODIFIER |
| c.222G>T | ORF1ab | synonymous_variant | p.Ser74Ser | LOW |
| c.318C>T | ORF1ab | synonymous_variant | p.Val106Val | LOW |
| c.393T>C | ORF1ab | synonymous_variant | p.Ala131Ala | LOW |
| c.619C>T | ORF1ab | missense_variant | p.Arg207Cys | MODERATE |
| c.898A>T | ORF1ab | missense_variant | p.Ile300Phe | MODERATE |
| c.1176C>T | ORF1ab | synonymous_variant | p.Gly392Gly | LOW |
| c.1878C>A | ORF1ab | synonymous_variant | p.Pro626Pro | LOW |
| c.1974A>C | ORF1ab | missense_variant | p.Glu658Asp | MODERATE |
| c.2672C>T | ORF1ab | missense_variant | p.Thr891Ile | MODERATE |
| c.3696C>T | ORF1ab | synonymous_variant | p.Ile1232Ile | LOW |
| c.3860T>C | ORF1ab | missense_variant | p.Val1287Ala | MODERATE |
| c.4317C>T | ORF1ab | synonymous_variant | p.Asn1439Asn | LOW |
| c.5435C>A | ORF1ab | missense_variant | p.Ala1812Asp | MODERATE |
| c.5465C>T | ORF1ab | missense_variant | p.Thr1822Ile | MODERATE |
| c.5805C>T | ORF1ab | synonymous_variant | p.Ile1935Ile | LOW |
| c.6260C>T | ORF1ab | missense_variant | p.Thr2087Ile | MODERATE |
| c.6710G>T | ORF1ab | missense_variant | p.Ser2237Ile | MODERATE |
| c.7075A>G | ORF1ab | missense_variant | p.Ile2359Val | MODERATE |
| c.8106G>T | ORF1ab | missense_variant | p.Gln2702His | MODERATE |
| c.8388G>T | ORF1ab | missense_variant | p.Met2796Ile | MODERATE |
| c.9319G>A | ORF1ab | missense_variant | p.Val3107Ile | MODERATE |
| c.10956G>T | ORF1ab | missense_variant | p.Met3652Ile | MODERATE |
| c.11604A>G | ORF1ab | synonymous_variant | p.Thr3868Thr | LOW |
| c.12138C>T | ORF1ab | synonymous_variant | p.Asn4046Asn | LOW |
| c.13611C>T | ORF1ab | synonymous_variant | p.Asp4537Asp | LOW |
| c.13842T>C | ORF1ab | synonymous_variant | p.Tyr4614Tyr | LOW |
| c.14113C>T | ORF1ab | synonymous_variant | p.Leu4705Leu | LOW |
| c.14404G>A | ORF1ab | missense_variant | p.Val4802Ile | MODERATE |
| c.15030C>T | ORF1ab | synonymous_variant | p.His5010His | LOW |
| c.15658G>A | ORF1ab | missense_variant | p.Gly5220Ser | MODERATE |
| c.16703C>T | ORF1ab | missense_variant | p.Thr5568Ile | MODERATE |
| c.16842T>C | ORF1ab | synonymous_variant | p.Gly5614Gly | LOW |
| c.18533G>T | ORF1ab | missense_variant | p.Gly6178Val | MODERATE |
| c.19757G>T | ORF1ab | missense_variant | p.Gly6586Val | MODERATE |
| c.19810A>T | ORF1ab | missense_variant | p.Ser6604Cys | MODERATE |
| c.20020G>T | ORF1ab | missense_variant | p.Ala6674Ser | MODERATE |
| c.249C>T | S | synonymous_variant | p.Val83Val | LOW |
| c.432T>C | S | synonymous_variant | p.Tyr144Tyr | LOW |
| c.764C>T | S | missense_variant | p.Ser255Phe | MODERATE |
| c.984A>G | S | synonymous_variant | p.Arg328Arg | LOW |
| c.1425C>T | S | synonymous_variant | p.Ala475Ala | LOW |
| c.2001T>C | S | synonymous_variant | p.Gly667Gly | LOW |
| c.2031G>T | S | missense_variant | p.Gln677His | MODERATE |
| c.2631G>T | S | synonymous_variant | p.Leu877Leu | LOW |
| c.2812C>A | S | missense_variant | p.Leu938Ile | MODERATE |
| c.3189G>T | S | missense_variant | p.Leu1063Phe | MODERATE |
| c.252G>T | ORF3a | synonymous_variant | p.Leu84Leu | LOW |
| c.273C>T | ORF3a | synonymous_variant | p.Tyr91Tyr | LOW |
| c.588A>C | ORF3a | synonymous_variant | p.Gly196Gly | LOW |
| c.673G>C | ORF3a | missense_variant | p.Val225Leu | MODERATE |
| c.13G>T | E | missense_variant | p.Val5Phe | MODERATE |
| c.5C>T | M | missense_variant | p.Ala2Val | MODERATE |
| c.123C>T | M | synonymous_variant | p.Asn41Asn | LOW |
| c.91T>C | ORF7a | synonymous_variant | p.Leu31Leu | LOW |
| c.116C>T | ORF7a | missense_variant | p.Thr39Ile | MODERATE |
| c.338G>T | ORF7a | missense_variant | p.Cys113Phe | MODERATE |
| c.108G>C | ORF8 | synonymous_variant | p.Pro36Pro | LOW |
| c.471C>T | N | synonymous_variant | p.Ile157Ile | LOW |
| c.497C>T | N | missense_variant | p.Thr166Ile | MODERATE |
| c.*63G>A | ORF10 | downstream_gene_variant |  | MODIFIER |
| n.29859T>A | ORF10-CHR_END | intergenic_region |  | MODIFIER |
| n.29868G>T | ORF10-CHR_END | intergenic_region |  | MODIFIER |

Supplementary Table 6: Extreme UVindex Specific rcntSNVs

| **rcntSNVs** | **Gene** | **Functional_rcntEffs** | **A.Acid-Change** | **rcntSNV_Impact** |
| --- | --- | --- | --- | --- |
| n.26A>G | CHR_START-ORF1ab | intergenic_region |  | MODIFIER |
| c.558C>T | ORF1ab | synonymous_variant | p.Val186Val | LOW |
| c.657G>A | ORF1ab | synonymous_variant | p.Leu219Leu | LOW |
| c.902G>A | ORF1ab | missense_variant | p.Arg301Gln | MODERATE |
| c.1185C>T | ORF1ab | synonymous_variant | p.Thr395Thr | LOW |
| c.1578G>T | ORF1ab | missense_variant | p.Gln526His | MODERATE |
| c.2247A>G | ORF1ab | synonymous_variant | p.Thr749Thr | LOW |
| c.2760G>T | ORF1ab | missense_variant | p.Met920Ile | MODERATE |
| c.2801G>A | ORF1ab | missense_variant | p.Gly934Asp | MODERATE |
| c.3528T>C | ORF1ab | synonymous_variant | p.Ala1176Ala | LOW |
| c.4391G>A | ORF1ab | missense_variant | p.Arg1464Gln | MODERATE |
| c.4621C>T | ORF1ab | missense_variant | p.Pro1541Ser | MODERATE |
| c.4910C>T | ORF1ab | missense_variant | p.Thr1637Ile | MODERATE |
| c.5848C>T | ORF1ab | missense_variant | p.Pro1950Ser | MODERATE |
| c.5946A>C | ORF1ab | synonymous_variant | p.Gly1982Gly | LOW |
| c.5948C>T | ORF1ab | missense_variant | p.Ala1983Val | MODERATE |
| c.6184C>T | ORF1ab | missense_variant | p.Leu2062Phe | MODERATE |
| c.8652C>T | ORF1ab | synonymous_variant | p.Phe2884Phe | LOW |
| c.8958C>T | ORF1ab | synonymous_variant | p.His2986His | LOW |
| c.9124G>A | ORF1ab | missense_variant | p.Asp3042Asn | MODERATE |
| c.9538C>T | ORF1ab | synonymous_variant | p.Leu3180Leu | LOW |
| c.10965G>T | ORF1ab | missense_variant | p.Met3655Ile | MODERATE |
| c.11388C>T | ORF1ab | synonymous_variant | p.Leu3796Leu | LOW |
| c.11676C>T | ORF1ab | synonymous_variant | p.Val3892Val | LOW |
| c.12873C>T | ORF1ab | synonymous_variant | p.Ile4291Ile | LOW |
| c.13240G>A | ORF1ab | missense_variant | p.Ala4414Thr | MODERATE |
| c.13303T>C | ORF1ab | missense_variant | p.Tyr4435His | MODERATE |
| c.14122G>T | ORF1ab | missense_variant | p.Ala4708Ser | MODERATE |
| c.15286G>T | ORF1ab | missense_variant | p.Ala5096Ser | MODERATE |
| c.15944C>T | ORF1ab | missense_variant | p.Pro5315Leu | MODERATE |
| c.16501C>T | ORF1ab | missense_variant | p.Pro5501Ser | MODERATE |
| c.17780C>T | ORF1ab | missense_variant | p.Ala5927Val | MODERATE |
| c.17811C>T | ORF1ab | synonymous_variant | p.Leu5937Leu | LOW |
| c.18187T>C | ORF1ab | missense_variant | p.Phe6063Leu | MODERATE |
| c.18219A>G | ORF1ab | synonymous_variant | p.Gly6073Gly | LOW |
| c.18933A>G | ORF1ab | synonymous_variant | p.Val6311Val | LOW |
| c.19647T>G | ORF1ab | synonymous_variant | p.Ala6549Ala | LOW |
| c.19750G>T | ORF1ab | missense_variant | p.Val6584Phe | MODERATE |
| c.20993C>T | ORF1ab | missense_variant | p.Thr6998Ile | MODERATE |
| c.842A>T | S | missense_variant | p.Glu281Val | MODERATE |
| c.882C>T | S | synonymous_variant | p.Asp294Asp | LOW |
| c.888C>T | S | synonymous_variant | p.Leu296Leu | LOW |
| c.1125C>T | S | synonymous_variant | p.Ser375Ser | LOW |
| c.3364G>T | S | missense_variant | p.Val1122Leu | MODERATE |
| c.3484C>T | S | missense_variant | p.Pro1162Ser | MODERATE |
| c.378G>T | ORF3a | missense_variant | p.Arg126Ser | MODERATE |
| c.8A>G | M | missense_variant | p.Asp3Gly | MODERATE |
| c.-6C>T | ORF6 | upstream_gene_variant |  | MODIFIER |
| c.98T>C | ORF6 | missense_variant | p.Ile33Thr | MODERATE |
| c.184G>T | ORF8 | missense_variant | p.Val62Leu | MODERATE |
| c.27G>T | N | missense_variant | p.Gln9His | MODERATE |
| c.62C>T | N | missense_variant | p.Ser21Leu | MODERATE |
| c.122G>T | N | missense_variant | p.Arg41Leu | MODERATE |
| c.478C>A | N | missense_variant | p.Gln160Lys | MODERATE |
| c.605G>A | N | missense_variant | p.Ser202Asn | MODERATE |
| c.626G>T | N | missense_variant | p.Arg209Ile | MODERATE |
| c.875T>C | N | missense_variant | p.Ile292Thr | MODERATE |
| c.*68G>A | ORF10 | downstream_gene_variant |  | MODIFIER |

Supplementary Table 7: Detailed number of total rcntSNVs in different gene’s groups for all UVindex categories

|  | **Gene based rcntSNVs distribution** | | | | | | | | | | |
| --- | --- | --- | --- | --- | --- | --- | --- | --- | --- | --- | --- |
| **UVindex Categories** | **Structural genes** | | | | **NSP genes** | **Accessory protein encoding genes** | | | | | |
|  | N | S | M | E | ORF1ab | ORF3a | ORF10 | ORF8 | ORF7b | ORF7a | ORF6 |
| **EXTREME** | 12 | 8 | 4 | 1 | 45 | 2 | 2 | 5 | 0 | 1 | 2 |
| **VERY_HIGH** | 7 | 14 | 3 | 2 | 47 | 6 | 2 | 2 | 2 | 3 | 2 |
| **HIGH** | 24 | 18 | 6 | 2 | 78 | 5 | 5 | 8 | 4 | 4 | 2 |
| **MODERATE** | 19 | 10 | 5 | 2 | 72 | 8 | 4 | 7 | 3 | 0 | 1 |
| **LOW** | 13 | 14 | 2 | 2 | 60 | 9 | 3 | 3 | 0 | 0 | 0 |

Supplementary Table 8: Commonly shared rcntSNVs among all five-UVindex categories (rcntSNVs near to fixation).

| **rcntSNVs** | **Gene** | **Funtional_rcntEffs** | **A.acid-Change** | **rcntSNV_Impact** |
| --- | --- | --- | --- | --- |
| c.2772C>T | ORF1ab | synonymous_variant | p.Phe924Phe | LOW |
| c.14159C>T | ORF1ab | missense_variant | p.Pro4720Leu | MODERATE |
| c.1841A>G | S | missense_variant | p.Asp614Gly | MODERATE |
| c.171G>T | ORF3a | missense_variant | p.Gln57His | MODERATE |
| c.608G>A | N | missense_variant | p.Arg203Lys | MODERATE |
| c.609G>A | N | synonymous_variant | p.Arg203Arg | LOW |
| c.610G>C | N | missense_variant | p.Gly204Arg | MODERATE |
